# Evaluating bone mineral density in osteoporotic vertebral compression fractures: the clinical utility of anterior column Hounsfield units

**DOI:** 10.3389/fendo.2025.1552780

**Published:** 2025-03-20

**Authors:** Jiabao Chen, Han Zheng, Haotian Li, Qingsong Yu, Yanhong Li, Huangda An, Lei Ma

**Affiliations:** ^1^ Department of Spinal Surgery, The Third Hospital of Hebei Medical University, Shijiazhuang, China; ^2^ Department of cardiology, Hebei General Hospital, Shijiazhuang, China

**Keywords:** Hounsfield unit, osteoporosis, bone mineral density, osteoporotic vertebral compression fractures (OVCFs), spinal surgery

## Abstract

**Study Design:**

Retrospective radiological analysis.

**Objective:**

This study aimed to evaluate the clinical utility of anterior column Hounsfield units (HU) in assessing bone mineral density (BMD) in patients with osteoporotic vertebral compression fractures (OVCFs) and to investigate its potential advantages over traditional measurement methods.

**Method:**

In this retrospective study, we analyzed data from 106 patients with acute OVCFs treated between January 2020 and June 2024. Inclusion criteria encompassed single-segment fractures from T10 to L2, with clear imaging results. HU values were measured from computed tomography (CT) scans, specifically targeting the anterior column of the vertebral body. Interobserver reliability was assessed via intraclass correlation coefficients (ICCs). Correlations between HU values, dual-energy X-ray absorptiometry (DEXA) results, and vertebral compression degrees were analyzed using Pearson correlation and receiver operating characteristic (ROC) curve analysis.

**Results:**

The average HU values were significantly lower in the anterior column (50.39 ± 21.62 HU) compared to the middle column (63.12 ± 25.14 HU). The anterior column HU values showed a strong positive correlation with DEXA T-scores (r = 0.643) and BMD (r = 0.656). The degree of vertebral compression also correlated positively with both HU values and DEXA results, with the anterior column HU demonstrating the highest correlation (r = 0.727). ROC analysis indicated that the anterior column HU value had the largest area under the curve (AUC = 0.913) for predicting severe OVCFs, with an optimal cutoff of 59.07 HU.

**Conclusion:**

The anterior column HU value serves as a superior predictor of BMD in patients with OVCFs compared to traditional methods. This study highlights the potential of using anterior column HU measurements to guide clinical decision-making regarding treatment options for OVCF patients, suggesting a shift towards more nuanced assessment strategies in osteoporosis management. Further research with larger sample sizes is warranted to validate these findings and explore the comprehensive application of HU values in osteoporosis evaluation.

## Introduction

Osteoporosis is characterized by a decrease in bone density for various reasons, along with the destruction of bone microstructure, which leads to increased bone fragility. Dual - energy X - ray absorptiometry (DXA) is currently the gold standard for quantifying bone mineral density and diagnosing osteoporosis. However, it still has certain limitations in evaluating spinal bone (1). In recent years, the measurement of Hounsfield unit (HU) by computed tomography (CT) has become an accepted technique for assessing bone quality. Previous studies have demonstrated that the HU value is closely associated with bone mineral density and the compressive strength of bone (2–7) HU values have been widely utilized in osteoporosis assessment, with the advantage of providing bone mineral density (BMD) data within the vertebrae. The CT HU value of the middle - axial image of the vertebral body is widely applied in clinical practice. It has high clinical value in predicting cage settlement and evaluating the pedicle screw holding force and may even be superior to the DEXA T - score (8–10).

For patients with vertebral compression fractures, it is very necessary to evaluate bone quality in order to guide the next step of treatment. Zou et al. (8) discovered that the L1 - HU value can serve as an excellent predictor of vertebral compression fractures. In their study, the average L1 - HU value in patients with acute vertebral fragility fractures was 66.0 HU. Nevertheless, this measurement fails to consider the uneven distribution of BMD within the vertebral body. Given the complexity of the spinal structure and the uniqueness of the load, the distribution of BMD in the vertebral bone is non – uniform (11).

In the majority of patients with osteoporotic vertebral compression fractures (OVCFs), the fracture typically occurs in the anterior column of the vertebral body (12, 13). Consequently, this study enhanced the measurement method for the CT HU value of the vertebral body. The HU value of the anterior column of the vertebral body was collected to explore its potential greater value for BMD assessment in OVCF patients.

The objectives of this study were twofold: first, to demonstrate the feasibility of the anterior - column CT HU value in evaluating the BMD of the thoracolumbar spines; second, to investigate the clinical application and advantages of the anterior - column CT HU value.

## Methods

### Patient cohort

This study received approval from the Institutional Review Board of our hospital. All patient data were retrospectively retrieved from the hospital’s medical record system. We examined the files of patients who had been treated for OVCFs in our department between January 2020 and June 2024.

#### Inclusion criteria

1.Acute vertebral compression fracture resulting from low - energy trauma, involving a single segment within T10 - L2. 2.Clear MRI, X - ray, CT, and DEXA examinations were obtainable. 3.The morphology of the vertebral body adjacent to the fractured vertebra was normal.

#### Exclusion criteria

1.Multilevel vertebral compression fracture. 2.Vertebral compression fractures induced by high - energy trauma. 3.Pathological fracture. 4.History of spinal surgery. 5.Kummel disease or diffuse idiopathic skeletal hyperostosis (DISH) morphology. 6.Long - term use of glucocorticoids.

### Date collection and assessment

The demographic data of the patients, such as gender, age, body mass index (BMI), and DEXA results, were recorded. Vertebral compression fractures were classified according to the method proposed by Genant (14). The imaging data were measured by two spine surgeons who had over three years of experience in imaging measurements. The HU measurement was obtained using a protocol similar to that described by Schreiber in CT examination.

All subjects were scanned with a 64 slice multi-detector CT scanner (Siemens Sensation 64, Erlangen, Germany) according to the following parameters: slice thickness 1.5 mm, distance 1.5 mm, tube voltage 120 kV. HU measurements were obtained from PACS (Picture Archiving and Communication Systems) Imaging System for lumbar vertebra.

**Figure 1:**
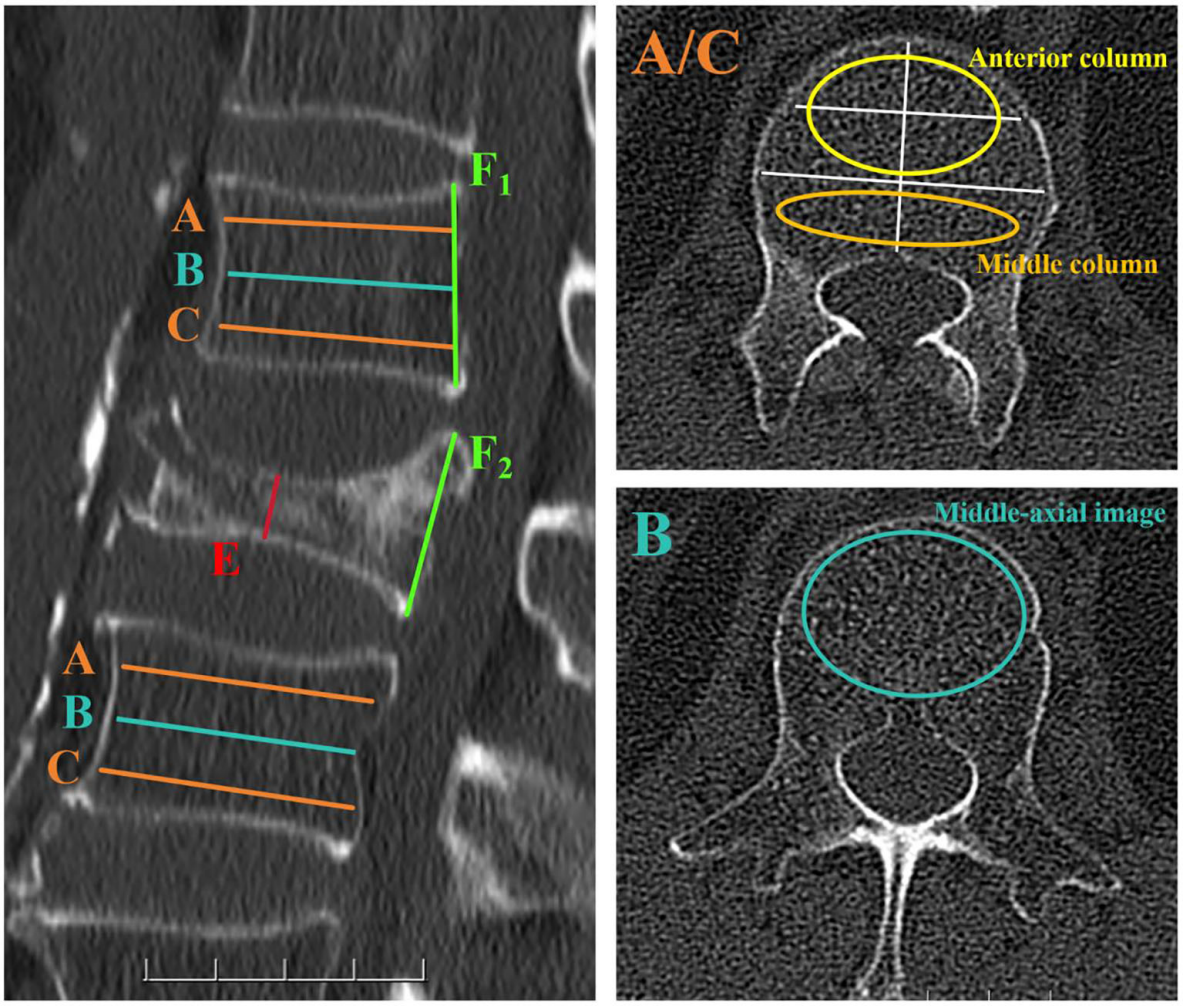
Computed tomography (CT) scan demonstrating the method for determining the HU value using an elliptical ROI. In the left - hand image, the axial slices of interest are shown on a sagittal slice of a CT scan of the lumbar vertebra. Slice A was obtained inferior to the superior end plate, while slice C was taken superior to the inferior end plate. Slice B represented the middle - axial image of the vertebral body. For slices A and C, two distinct locations on each axial slice were selected as ROIs for HU measurements: the anterior two - thirds of the vertebrae and the posterior one - third of the vertebrae. For slice B, a large elliptical area was used as an ROI to measure HU values. Elliptical ROIs were drawn as large as possible, excluding cortical edges to prevent volume averaging. The compression ratio of osteoporotic vertebral compression fractures was determined by E/F1. If no compression occurred at the posterior edge of the fractured vertebra, E/F2 was used to determine the compression ratio.

For the vertebrae with compression fractures, two adjacent vertebrae were selected for the measurement of HU values. Three different axial sections in each vertebral body were chosen, namely immediately below the superior end plate, at the mid - vertebral body, and above the inferior end plate. At the mid - axial image, HU values were obtained following the methods described in previous studies (15). For the two axial slices close to the upper and lower end plates, two different locations in each axial slice were selected as regions of interest (ROI) for HU measurements: the anterior two - thirds of the vertebrae and the posterior one - third of the vertebrae. The ROI was designed to encompass as much trabecular bone as possible while avoiding cortical bone and heterogeneous areas such as the posterior venous plexus and bone islands. The average of the HU values in the anterior two - thirds of the vertebral body represents the HU value of the anterior column of the vertebral body, and the average HU value in the posterior one - third of the vertebral body represents the HU value of the middle column of the vertebral body (**Figure 1**).

For osteoporotic vertebral compression fractures, the compression ratio is defined as the ratio of the height of the most compressed part of the vertebral body to that of the posterior edge of the vertebral body. In cases where whole - body compression has occurred, the compression ratio for osteoporotic vertebral compression fractures is the ratio of the height of the most compressed part of the vertebral body to the average height of the posterior edges of the upper and lower vertebral bodies.

### Statistical analysis

Data were analyzed using Statistical Product and Service Solutions software (version 26; SPSS, Chicago, IL). Continuous variables were documented as mean ± standard deviation. The interclass correlation coefficients (ICCs) were computed to appraise interobserver reliability. The Pearson correlation test was employed to analyze the correlation between the outcomes of different HU measurements and DEXA results. Receiver operating characteristic curve (ROC) analysis and the area under the curve (AUC) were utilized to assess the performance of using HU value and T - score in differentiating severe compression fractures.

## Results

### Patient characteristics

A total of 106 patients were enrolled in the study, including 22 males and 84 females. The mean age of the patients was 70.92 ± 7.878 years. There were 7 cases of T10 fracture, 22 cases of T11 fracture, 33 cases of T12 fracture, 30 cases of L1 fracture and 14 cases of L2 fracture. The degree of fracture compression was graded according to Genant classification, including 4 patients with grade 0, 7 patients with grade 1, 37 patients with grade 2 and 58 patients with grade 3. The mean BMD and T value of DEXA were 0.53 ± 0.10g/cm2 and -2.88 ± 0.81, respectively. The average HU value of the axial position was 67.35 ± 28.31HU, the average HU value of the anterior column was 50.39 ± 21.62HU, and the average HU value of the middle column was 63.12 ± 25.14HU. The average vertebral compression ratio was 0.48 ± 0.14 (**Tables 1**, **2**).

**Table 1:** Analysis of general data of 106 patients with OVCFs.

Variable	ALL (n=106)
Age	70.92 ± 7.878
Sex	
Male	22
Female	84
BMI	24.50 ± 2.76
Fractured vertebral body	
T10	7
T11	22
T12	33
L1	30
L2	14
Genant classification	
Grade 0	4
Grade 1	7
Grade 2	37
Grade 3	58
Femoral neck BMD	0.53 ± 0.10
Femoral neck T-score	-2.88 ± 0.81
HU value	
Anterior column	50.39 ± 21.62
Middle column	63.12 ± 25.14
Middle-axial image	67.35 ± 28.31
Vertebral compression ratio	0.48 ± 0.14

**Table 2:** Evaluation of BMD for different degrees of vertebral compression fractures.

		Grade0-2 (n=48)	Grade 3 (n=58)	ALL (n=106)
Femoral neck BMD		0.59 ± 0.09	0.49 ± 0.08	0.53 ± 0.10
Femoral neck T-score		-2.42 ± 0.71	-3.26 ± 0.68	-2.88 ± 0.81
HU Value	Anterior column	66.76 ± 14.20	36.85 ± 16.80	50.39 ± 21.62
	Middle column	81.01 ± 17.90	48.31 ± 20.18	63.12 ± 25.14
	Middle-axial image	85.22 ± 23.97	52.56 ± 22.61	67.35 ± 28.31

### Consistency test

The inter-rater reliability of measurements obtained by two spinal surgeons was assessed using the Interclass Correlation Coefficient (ICC), which exceeded 0.96 at each location (Five ROI in each vertebral body), indicating high agreement between the data measured. For the vertebral compression ratio, the ICC across the 106 cases for both observers was 0.975, which indicated high agreement between the data measured.

### Correlation between T - value/BMD value of DEXA and HU value of anterior and middle vertebral column

**Figure 2:**
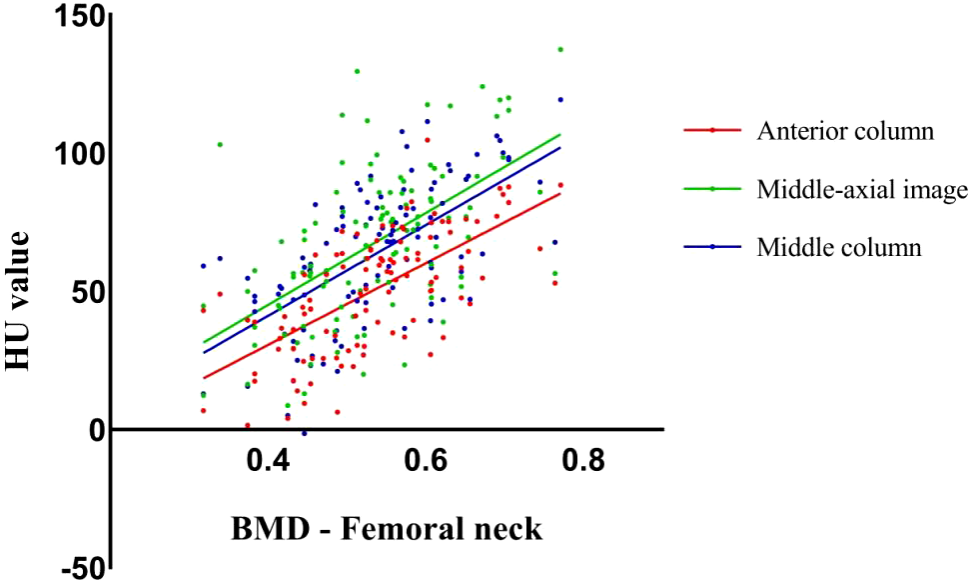
Scatter plots showed the correlation between CT HU values in different ROI of the vertebral body and bone mineral density scores obtained from DEXA of the femoral neck.

Both anterior and middle column CT HU values were positively correlated with the femoral neck T - score/BMD in DEXA results. (**Table 3**, **Figure 2**) The correlation coefficient between the HU value of anterior column and T-score (r = 0.643)/BMD (r = 0.656) was the highest, which was greater than that between the middle-axial image HU value and T - score (r = 0.555)/BMD (r = 0.564).

**Table 3:** Pearson correlation coefficients between CT HU values of different ROI of the vertebral body and femoral neck T-score or BMD in DEXA.

		Correlation coefficients	
		T-score	BMD
HU Value	Anterior column	0.643**	0.656**
	Middle column	0.609**	0.627**
	Middle-axial image	0.555**	0.564**

**P value<0.001.

### Correlation between vertebral compression degree and DEXA results as well as HU values

In patients with osteoporotic vertebral compression fractures, the degree of vertebral compression exhibited a positive correlation with both HU values and DEXA results. Notably, the HU value of the anterior column and the degree of vertebral compression demonstrated a relatively high Pearson correlation coefficient (r = 0.727) (**Table 4**).

**Table 4:** Pearson correlation coefficients between vertebral compression ratio and CT HU value/DEXA results.

		HU Value			T-score	BMD
		Anterior column	Middle column	Middle-axial image		
Vertebral compression ratio	Correlation coefficients	0.727	0.657	0.600	0.502	0.517
	*p*	0.000	0.000	0.000	0.000	0.000

### Identifying optimal predictors for severe osteoporotic fractures using ROC curves

**Figure 3:**
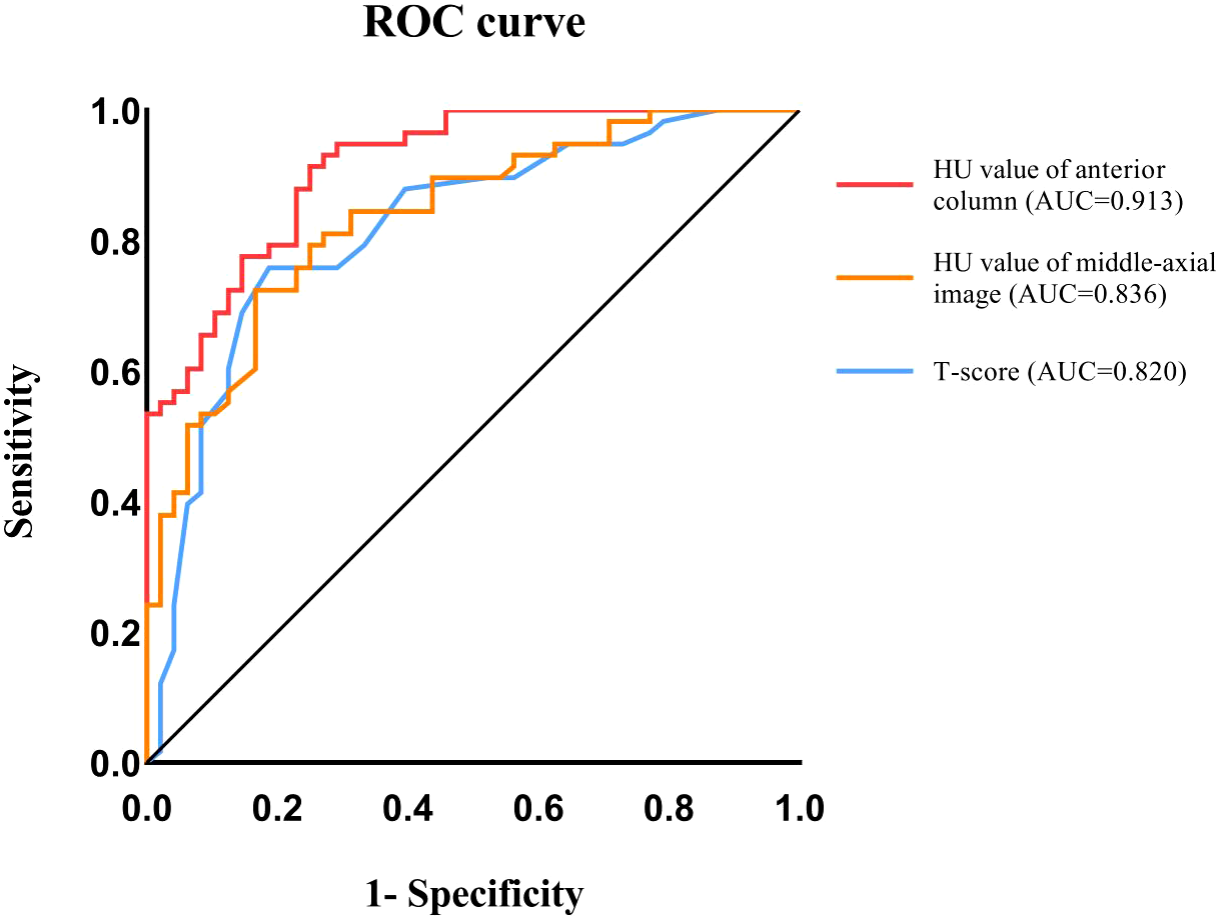
Receiver-operating characteristic curve (ROC) analysis was used to evaluate the performance of HU value and T-score in distinguishing severe compression fractures.

ROC curves were generated and the area under the curve (AUC) was measured to identify the optimal predictors for severe osteoporotic compression fractures (Grade 3). The results demonstrated that the ROC curve of the anterior column HU value had the largest AUC of 0.913. The Youden index was utilized to determine the optimal cutoff value of 59.07 HU, with a sensitivity of 0.75 and a specificity of 0.914. The AUC of the ROC curve of the HU value in the middle - axial image was 0.836, and the Youden index determined the optimum critical value to be 64.55 HU, with a sensitivity of 0.833 and a specificity of 0.724. The AUC of the ROC curve of the T - score was 0.820, and the Youden index determined the optimum critical value to be - 2.85, with a sensitivity of 0.813 and a specificity of 0.759 (**Figure 3**).

## Discussion

Osteoporosis frequently occurs in the elderly and postmenopausal women, thereby augmenting their fracture risk and imposing a burden on society. In accordance with national and international guidelines, bone quality should be evaluated by DEXA or quantitative computed tomography (qCT). DEXA, a non - invasive spectral imaging method, is regarded as the gold standard for diagnosing osteoporosis. Nevertheless, spinal degeneration might result in inaccurate BMD measurements when using this technique (16). Quantitative computed tomography can surmount these limitations. In qCT, HU are converted to BMD values via software using a standard CT scanner and additional reference standards. However, this technique is not the preferred recommendation of the World Health Organization (WHO) for BMD measurement and is not as extensively utilized as DEXA.

At present, the average HU value of the lumbar spine has been widely utilized in clinical practice. The common approach for HU measurement is to select the region of interest in the mid - axial image of the vertebral body. However, as per previous studies, the uneven distribution of bone mineral density within the vertebral body has been rarely taken into account. According to the Delpech - Wolff law, bone formation is influenced by mechanical stimuli. The distribution of pressure and tension molds the microstructure of the bone and promotes bone formation, thereby increasing bone density, and vice versa. The structure of the human spine is complex. The spine endures multi - dimensional stress and shear forces, and the load within the spine is highly complex; thus, the distribution of bone mineral density is also uneven.

In clinical practice, the uneven distribution of BMD in vertebrae is frequently overlooked. Consequently, the application and measurement of HU values require improvement. In 1984, Ferguson refined Denis’s three - column theory of the spine. Ferguson defined the anterior column as the anterior longitudinal ligament and the anterior two - thirds of the vertebral body and disc, and the middle column as the posterior longitudinal ligament and the posterior one - third of the vertebral body and disc. In this study, we enhanced the measurement method of HU values and selected the ROI at the anterior column of the vertebral body.

### Feasibility of anterior column HU value in evaluating vertebral BMD

In this study, the ROI for HU value measurement was selected differently from previous studies. Slices adjacent to the upper and lower endplates were chosen as the ROI to measure the HU value of the anterior column. Meanwhile, according to previous studies, the middle - axial image of the vertebral body was selected as the ROI for reference. The results demonstrated that both the HU values of the anterior column and those of the middle - axial image were significantly correlated with the DEXA results. This suggests that the anterior column HU value can serve as a reliable indicator for bone mineral density evaluation in OVCF patients.

Moreover, the HU values of the anterior column were more strongly correlated with the DEXA results than those of the middle - axial images. In light of this result, we supposed that there could be more blood vessels in the middle - axial image of the vertebral body, although the ROI did not include the vertebral basal vein foramen. In elderly patients with osteoporosis, uncalcified or calcified blood vessels in the vertebra may interfere with the measurement of HU values. Thus, HU values measured away from the middle - axial images are more strongly correlated with DEXA results.

### The utility of anterior column hu value in assessing vertebral bone mineral density

In the spinal structure, the intervertebral disc and facet joints exhibit a certain degree of motion during spinal flexion and extension. This causes the middle column of the spine to function analogously to the fulcrum of a seesaw. Consequently, relative to the anterior column of the vertebral body, the middle column bears more stress, leading to a higher bone mineral density. However, when a vertebral compression fracture occurs, it is typically a result of a relatively stronger load on the anterior column of the spine.

In this study, according to the three - column theory of the spine, not only were the HU values of the middle - axial image measured, but also the CT HU values of the anterior and middle columns of the vertebral body were measured. The results demonstrated that there was a significant difference between the average HU value of the anterior column and that of the middle - axial image, with the HU value of the anterior column being lower. In the T10 - L2 vertebral body, compared with other osteoporosis evaluation indicators, the HU value of the anterior column and the degree of vertebral compression exhibited a more obvious correlation. Moreover, the HU value of the anterior column has a higher predictive value for severe OVCF (grade 3), which may offer important information for the selection of treatment options for OVCF patients.

In previous clinical experiences, the majority of patients with OVCF are recommended to stay in absolute bed rest (ABR) for one to two months prior to getting out of bed. Nevertheless, long - term bed rest can result in a series of complications, including pneumonia, bedsore, urinary tract infection, deep vein thrombosis, and cerebral infarction. Even just one week of ABR can cause severe muscle atrophy and insulin resistance throughout the body (17). Moreover, bone formation ceases during ABR, leading to further bone loss. Previous studies have proposed that patients with OVCF might benefit from a short - term 3 - day ABR followed by bracing (18). However, this treatment has not been accepted by most clinicians yet.

In this study, we observed that the HU value in the middle column of the vertebra was substantially higher than that in the anterior column, signifying a greater BMD in the middle column. This surely offers a foundation for the short - term ARB treatment concept in OVCF patients. With the brace’s protection, the anterior column of the OVCF vertebral body can evade excessive stress, while the middle column can endure relatively more stress. Nevertheless, we also noted that patients with severe OVCF (Grade 3) had an average anterior - column HU of merely 36.85, implying that patients with short - term ARB might be at a higher risk of further vertebral compression.

ROC curves were employed to analyze the predictive value of these osteoporosis measures for severe OVCFs. The results demonstrate that the anterior column HU value has the highest AUC, indicating a higher predictive value for severe OVCFs. The AUC of the ROC curve for the anterior - column HU value was 0.913, and the optimal cut - off value, determined by the Youden index, was 59.07 HU, with a specificity of 0.9140. The results suggest that when the HU value of the anterior column of the vertebral body is greater than 59.07, severe vertebral compression fracture is less likely to occur upon the vertebral body being subjected to low - energy trauma.

To the best of our knowledge, no studies have yet identified a suitable BMD evaluation index for assessing whether OVCF patients are suitable for short - term ABR. This study offers insights for subsequent clinical diagnosis and treatment, and the anterior - column HU value can serve as a favorable BMD evaluation index to guide patients towards further treatment. We look forward to future studies further validating these results and exploring the broader application of HU values in osteoporosis assessment.

## Limitations

Although the results of this study provide new insights for clinical practice, there are still some limitations: 1.In this study, strict inclusion criteria were adopted for restriction, so the sample size of patients was small, and studies with a larger sample size are still required for verification. 2.Previous studies have shown that vertebral compression fractures are related to other factors such as lumbar muscle strength, but similar parameters were not included in this study. 3.This study only conducted research on patients with OVCFs, lacking the comparison of the anterior column HU values in non - OVCFs patients. 4.This study did not explore the comprehensive application of HU values and other osteoporosis - related indicators, and multi - index combined evaluation can be considered in the future.

## Data Availability

The raw data supporting the conclusions of this article will be made available by the authors, without undue reservation.
